# Inspection of Imported Drugs

**Published:** 1848-07

**Authors:** 


*Inspection of Imported Drugs.*—The bill providing for the inspection of imported drugs has passed the Senate, and is now a law. We trust it will be faithfully executed.

